# M6A plays a potential role in carotid atherosclerosis by modulating immune cell modification and regulating aging-related genes

**DOI:** 10.1038/s41598-023-50557-8

**Published:** 2024-01-02

**Authors:** Wenpeng Zhao, Yingqi Xu, Jiabao Zhu, Chaoxuan Zhang, Weimin Zhou, Shizhi Wang

**Affiliations:** 1 Department of Vascular Surgery, The Second Affiliated Hospital of Nanchang University, Jiangxi Medical College, Nanchang University, No. 1 Minde Road, Nanchang, 330006 Jiangxi Province China; 2 Queen Mary College, Nanchang University, Nanchang, 330031 Jiangxi China

**Keywords:** Molecular biology, Cardiovascular biology

## Abstract

RNA N6-methyladenosine (m6A) regulators play essential roles in diverse biological processes, including immune responses. Mounting evidence suggests that their dysregulation is intricately linked to numerous diseases. However, the role of m6A-associated genes in carotid atherosclerosis and their relationship with aging and immune cells remain unclear. Analyze the expression profiles of m6A-related genes in carotid atherosclerosis-related datasets. Based on the expression patterns of m6A-related genes, perform consistent clustering analysis of carotid atherosclerosis samples and investigate associated immune cell infiltration patterns and aging characteristics. Develop an m6A prediction model specific to carotid atherosclerosis and analyze the relationships between immune cells infiltration and aging features. The m6A methylation modification level exhibited a substantial decrease in early-stage carotid atherosclerosis samples compared to late-stage carotid atherosclerosis samples. Subsequently, two distinct m6A subtypes were defined through consensus clustering analysis, with the lower m6A modification level group showing associations with heightened immune cell infiltration and increased expression of aging-related genes. A model composed of five m6A-related genes was formulated, and the results indicated that this model possesses effective predictive and therapeutic capabilities for carotid atherosclerosis. Furthermore, the downregulation of YTHDC1 expression resulted in elevated expression of inflammatory factors and a decrease in the expression of the aging-related gene RGN. Single-cell data analysis suggests that the reduced expression of YTHDC1 may decrease the degradation of inflammation-related factors in macrophages, leading to a highly inflammatory state in the carotid artery wall. Furthermore, the sustained release of inflammatory factors may increase the expression of the aging-related gene RGN in vascular smooth muscle cells, further exacerbating the progression of atherosclerosis. A reduced level of m6A methylation modification could enhance inflammation and expedite cellular aging, thereby contributing to the development of carotid atherosclerosis.

## Introduction

According to World Health Organization data for 2011, approximately 13.2 million individuals succumbed to cardiovascular diseases (CVD), representing 24% of the total global mortality. Projections indicate that by 2030, the worldwide mortality attributable to CVD may escalate to as high as 23.3 million, affirming their continued status as the primary cause of disease-related fatalities^1^. Atherosclerosis, characterized as a chronic arterial disease, significantly contributes to the majority of cardiovascular diseases. Its principal pathological feature involves the gradual accumulation of lipid deposits within the arterial wall, ultimately leading to the development of atherosclerotic plaques and distinctive lesions. Notably, carotid atherosclerosis stands as a prominent instigator of cerebrovascular diseases. The progression of carotid atherosclerosis can culminate in either partial or complete constriction of the vascular lumen, resulting in diminished blood flow to the brain. This, in turn, can precipitate symptoms such as blurred vision and episodes of sudden loss of consciousness in affected individuals. Furthermore, a more critical consequence entails the acute rupture of carotid atherosclerotic plaques, potentially leading to the formation of local thrombi and subsequent blockages in the vasculature^2,3^. This constitutes a primary etiological factor for ischemic strokes and cerebral infarctions, and stroke ranking as the second most prevalent cause of global mortality^4,5^. While the incidence and mortality rates of carotid atherosclerosis have markedly diminished in high-income countries owing to enhanced living conditions, advancements in healthcare, and heightened attention to health management^6^, it persists as a formidable public health concern in numerous low- and middle-income countries.

In recent years, numerous studies have undertaken investigations into epigenetic modifications within the context of atherosclerosis. These investigations have revealed distinct features of acetylation, phosphorylation, and DNA methylation within endothelial cells, smooth muscle cells, and macrophages in individuals affected by atherosclerosis^7–9^. While DNA modifications and histone modifications have received extensive scrutiny within the field of epigenetics, our understanding of RNA modifications remains limited. In contrast to the relatively restricted scope of DNA epigenetic modifications, researchers have identified more than 100 different types of RNA modifications in molecules such as ribosomal RNA (rRNA), messenger RNA (mRNA), and transfer RNA (tRNA). These modifications perform pivotal roles in the regulation of RNA stability, translation, splicing, and cellular aging processes^10^. It is noteworthy that N6-methyladenosine (m6A) stands as one of the most prevalent and extensively researched chemical modifications within mRNA^11^. m6A methylation specifically pertains to methylation at the N6 position of adenosine nucleotides. Its primary functionality is orchestrated through three distinct categories of regulators, impacting facets such as RNA stability, aging, and translation. These categories consist of Writers, responsible for catalyzing methylation; Erasers, responsible for demethylation; and Readers, directly recognizing and binding to m6A sites, thus enabling m6A-modified RNA to execute specific functions^12^. m6A modification has been demonstrated to hold pivotal roles in various domains, including cancers, metabolic diseases, and cardiovascular diseases^13–15^. Nevertheless, current research is deficient in its exploration of m6A modification in the context of carotid atherosclerosis and its correlation with the immune microenvironment and aging characteristics.

In this study, three carotid atherosclerosis datasets were obtained from the Gene Expression Omnibus (GEO) database. Differential expression analysis of m6A-related genes in carotid atherosclerosis samples was conducted. An m6A-related gene model was established through random forest analysis, and consistent clustering analysis was performed to investigate m6A involvement in carotid atherosclerosis. Subsequently, the association between m6A modification and immune cell infiltration, as well as aging, was explored following the methodology outlined by Yoshihara et al.^16^. The aim was to identify m6A genes that play a pivotal role in the onset of carotid atherosclerosis.

## Materials and methods

### Data collection and processing

Gene expression data for carotid atherosclerosis were obtained from the GSE43292; GSE28829; GSE100927; GSE159677 datasets^17–20^ in the Gene Expression Omnibus (GEO) database (https://www.ncbi.nlm.nih.gov/geo/). Among these, GSE43292 dataset contains 32 carotid artery plaque and 32 intact carotid tissue; GSE28829 dataset contains 13 early atherosclerotic plaques and 16 advanced atherosclerotic plaques; GSE100927 contains 29 carotid artery plaque and 12 carotid tissue; GSE159677 dataset contains 6 samples, including 3 calcified atherosclerotic core plaques and 3 control samples.

### Acquisition and differential analysis of m6A-related genes

m6A-related genes were compiled through an exhaustive literature review, encompassing: Writers (METTL3, METTL14, METTL16, WTAP, ZC3H13, RBM15, RBM15B, CBLL1), Erasers (ALKBH5, FTO), and Readers (YTHDC1, YTHDC2, YTHDF1, YTHDF2, YTHDF3, HNRNPC, FMR1, LRPPRC, HNRNPA2B1, IGFBP1, IGFBP2, IGFBP3, RBMX, ELAVL1, IGF2BP1, IGF2BP2, IGF2BP3). The "limma" software package^21^ in R software was used to extract and integrate data from diverse datasets containing m6A-related genes. Subsequently, differential analysis was conducted contingent on sample types. The positions of differentially expressed m6A genes on chromosomes were visualized using the "RCircos" package^22^.

### Clustering analysis and PCA analysis

Based on the expression profiles of the m6A genes, unsupervised consensus clustering analysis of the carotid atherosclerosis samples was performed using the "ConsensusClusterPlus" software package, resulting in the division of the samples into two distinct m6A clusters^23^. Principal component analysis (PCA) was employed to achieve dimensionality reduction.

### Immune cell infiltration in different clusters

The "GSVA" package^24^ was employed to assess the infiltration of 23 different types of immune cells in various clusters. Correlation analysis was conducted between the differentially expressed m6A-related genes and immune cells.

### DEGs between the two m6A clusters and functional analysis

The "limma" software package was used to analyze the differentially expressed genes (DEGs) between the two m6A clusters^21^. We used "clusterProflier 4.0" to perform GO (Gene Ontology) and KEGG (Kyoto Encyclopedia of Genes and Genomes) functional enrichment analyses to explore the potential biological functions of these DEGs^25^.

### Random forest (RF) and support vector machine (SVM) model construction

Model construction and comparison between the RF model and the machine learning method (SVM model) were undertaken. ROC analysis was performed to assess the predictive accuracy of both models using the pROC package and to calculate the area under the curve (AUC).

### Construction of nomogram model and evaluation

The "rms" package is used to construct nomograms. The decision curve analysis (DCA), clinical impact curves and the calibration curve based on model-related genes for carotid atherosclerosis samples to assess the effectiveness.

### Immune cell infiltration and aging scores in carotid atherosclerosis samples

Utilizing the "GSVA" package^24^, the infiltration of 23 different types of immune cells in samples of carotid atherosclerosis was assessed. A total of 307 aging-related genes (AG) were sourced from the HAGR database (http://genomics.senescence.info/genes/). Differential expression analysis of AGs in carotid atherosclerosis samples was conducted, and AG scores within carotid atherosclerosis samples and across m6A clusters were assessed using the "GSVA" package^24^.

### Correlation between m6A model genes and immune cell infiltration and aging characteristics

The correlation between gene expression differences in the m6A model and the infiltration of 23 types of immune cells, as well as the correlation with aging-related genes (AG), was evaluated.

### Single-cell data analysis

We utilized the "seurat" package for the organization of single-cell data. The samples consisted of 3 calcified atherosclerotic core (AC) plaques and 3 proximal adjacent (PA) portions of carotid artery. Cells with gene expression greater than 200 and less than 5000 were retained, and cells with a mitochondrial percentage greater than 20% were filtered out. A total of 48,292 cells were included in the analysis, and the top 2000 highly variable genes were selected for sample integration. Clustering analysis of cells was then performed with a resolution of 0.5. Visual representation of cell clusters was achieved using Uniform Manifold Approximation and Projection (UMAP). Differential expression analysis was employed to showcase the top genes for each cluster. Additionally, cell classification based on relevant marks was conducted for the clusters.

### Statistical analysis

All analyses were conducted using R software (version 4.1.0). Spearman and distance correlation analyses were employed to estimate correlation coefficients between the expression of m6A regulators and infiltrating immune cells. The Wilcoxon test was performed to assess variations between two groups. A ROC curve was generated to validate the model's efficacy. Statistical significance was defined as P < 0.05.

## Results

### Expression profile of m6A regulatory factors in atherosclerosis samples

**Figure 1:**
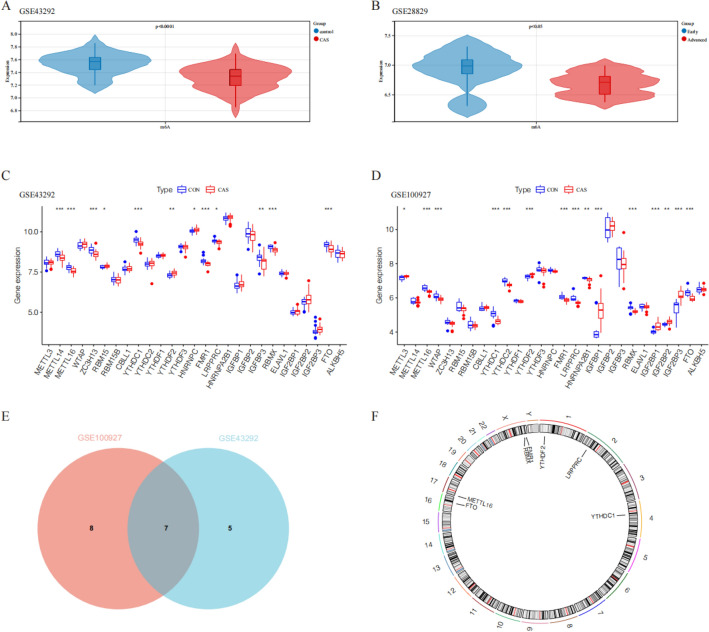
The expression and distribution of m6A regulatory factors in CAS. Box plots depicting the expression of 27 m6A regulatory factors in normal and CAS groups (**A**) early-stage CAS and late-stage CAS groups (**B**). Box plots illustrating the expression of 27 m6A regulatory factors in the GSE43292 and GSE100927 datasets (**C**, **D**). (**E**) Box plots illustrating the expression of 27 m6A regulatory factors in the GSE43292 and GSE100927 datasets. (**F**) Location of variations in m6A regulatory factors on 22 chromosomes. *P < 0.05; **P < 0.01; ***P < 0.001.

In this study, a total of 27 m6A regulatory factors were examined, consisting of 8 Writers, 2 Erasers, and 17 Readers. By analyzing the expression profiles of these m6A regulatory factors in the GSE43292 and GSE100927 datasets, it was observed that compared to control samples, carotid artery plaque samples exhibit a tendency of decreased total m6A methylation scores (Fig. 1A,B), and gene expression details between carotid artery plaque group and control group are shown in Fig. 1C,D. We found that the RBMX, FMR1, METTL16, YTHDC1, FTO, YTHDF2 and LRPPRCA m6A regulatory factors exhibited statistical significance in both datasets (Fig. 1E). Finally, the chromosomal positions of these 7 m6A regulatory factors were summarized (Fig. 1F).

### Two modes of m6A RNA methylation mediated by 26 regulatory factors

**Figure 2:**
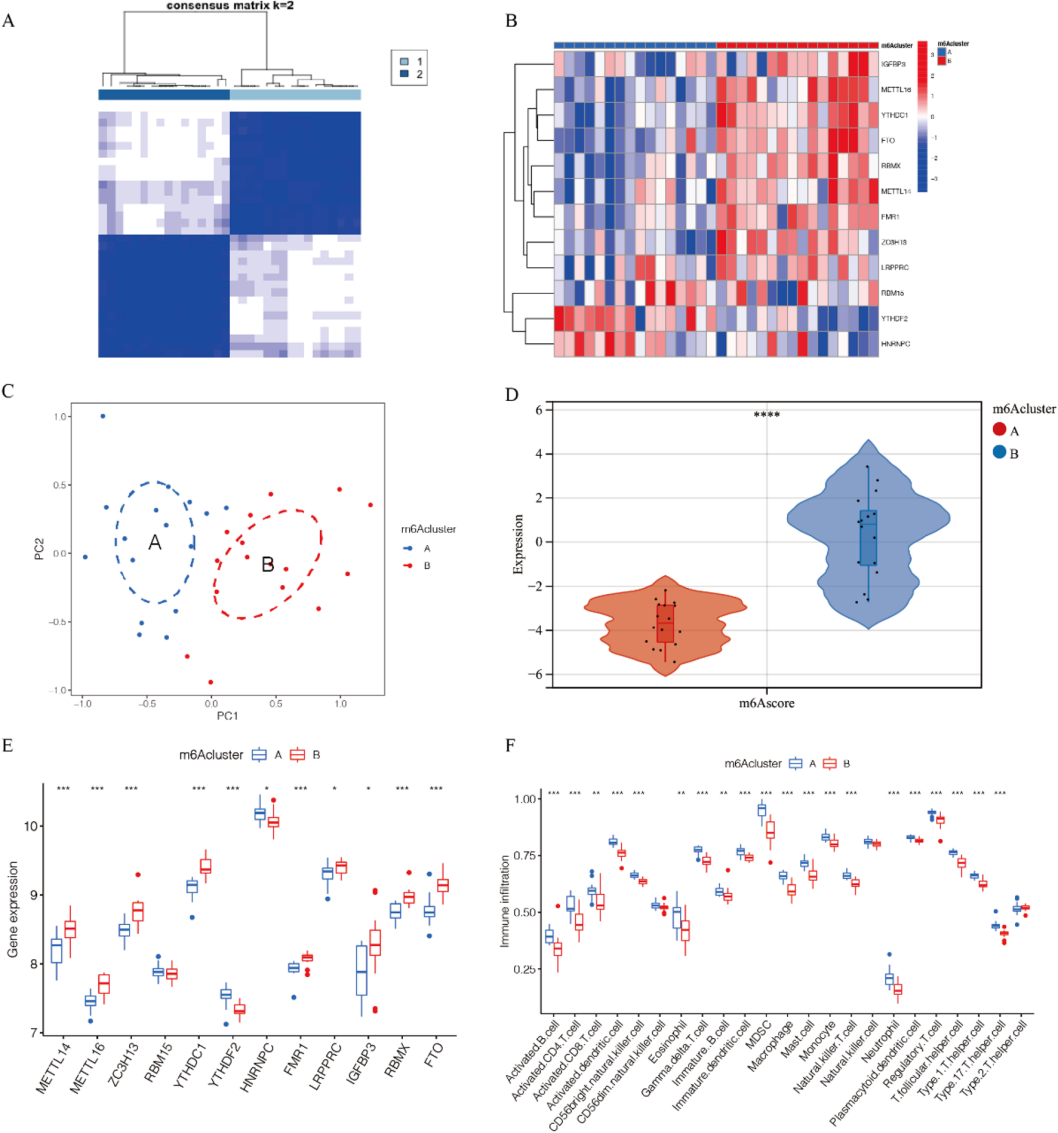
Consensus clustering Analysis and PCA Analysis of m6A regulatory factors. (**A**) Heatmap displaying the Matrix of co-occurrence percentages in CAS samples. (**B**) Expression of m6A-related genes in different clusters. (**C**) Principal Component Analysis (PCA) plot. (**D**, **E**) Expression profiles of m6A regulatory factors under A and B patterns. (**F**) Immune cell infiltration of m6A regulatory factors between samples under A and B patterns. *P < 0.05; **P < 0.01; ***P < 0.001.

**Figure 3:**
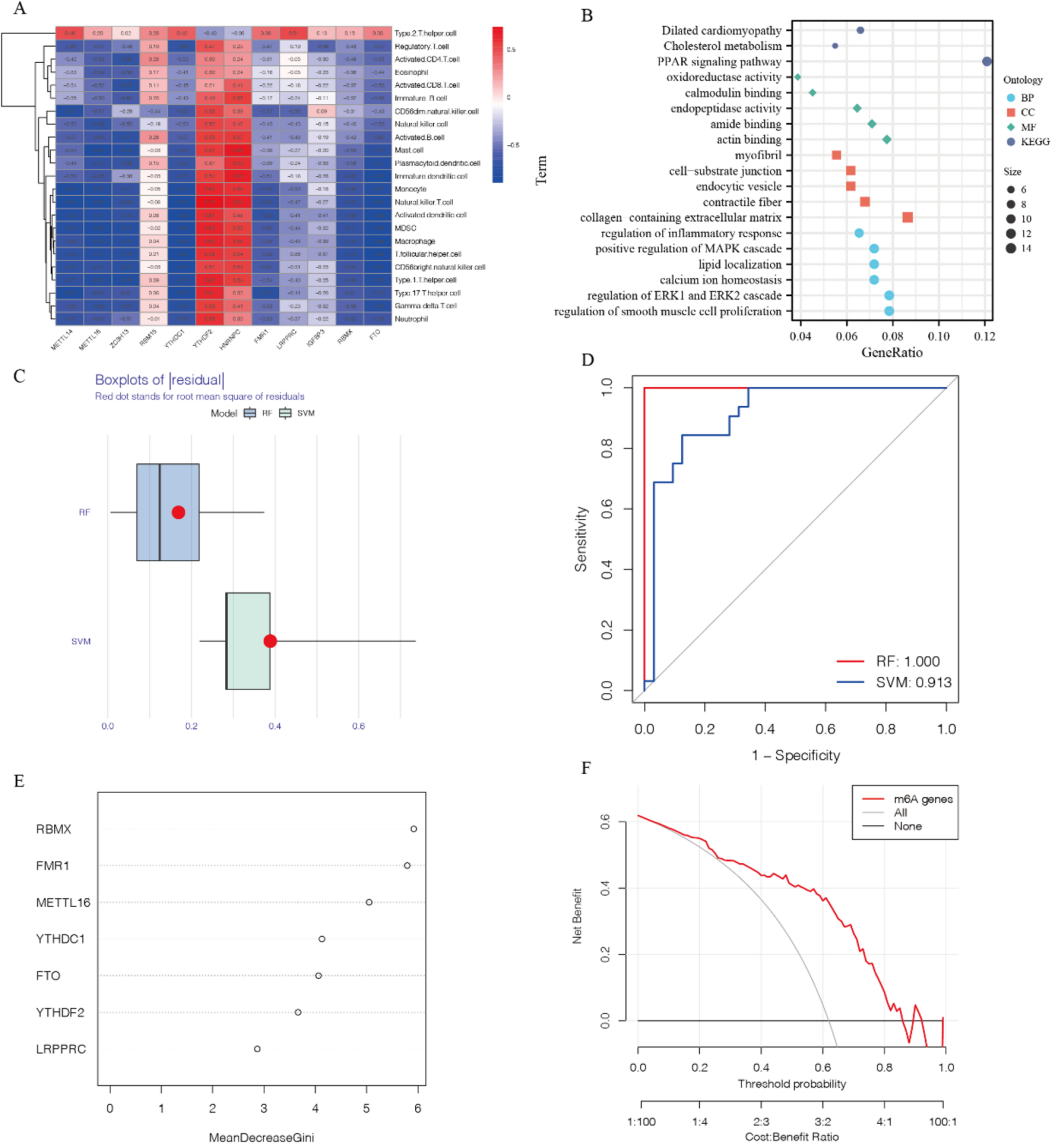
Construction of the random forest (RF) model and support vector machine (SVM) model. (**A**) Heatmap illustrating the correlation between 12 m6A regulatory factors and immune cells. (**B**) Functional analysis and KEGG pathway analysis of m6A regulatory factors. (**C**) Box plots depicting the residuals of SVM and RF models. (**D**) ROC curves of SVM and RF models. (**E**) Importance score of key m6A regulatory factors in the RF model. (**F**) Decision Curve Analysis (DCA) curves.

Based on the expression levels of m6A regulatory factors, we performed unsupervised consensus clustering analysis on carotid atherosclerosis samples. The results indicate that the optimal clustering outcome was achieved when K = 2 (Fig. S1A,B and Fig. 2A,B). Additionally, we conducted principal component analysis (PCA), which revealed that CAS patients can be divided into two clusters based on m6A regulatory factors (Fig. 2C). Furthermore, as shown in Fig. 2D,E, compared to cluster B, cluster A exhibited lower expression. In addition, compared to cluster B, cluster A shows more immune cell infiltration (Fig. 2F). Correlation analysis indicates that YTHDF2 has the strongest positive correlation with most immune cells, especially NK cells, macrophages, and helper T cells. In contrast, YTHDC1 is negatively correlated with most immune cells, especially macrophages and helper T cells (Fig. 3A). It may indicate that low expression of m6A-related genes is associated with more recruitment of immune cells. Furthermore, considering the relationship between m6A modification and gene stability and degradation, we speculate that the decrease in m6A-related gene expression may lead to a decrease in the degradation of inflammatory factors, thus triggering a more severe inflammatory response.

### Identification of key m6A regulatory factors in CAS

**Figure 4:**
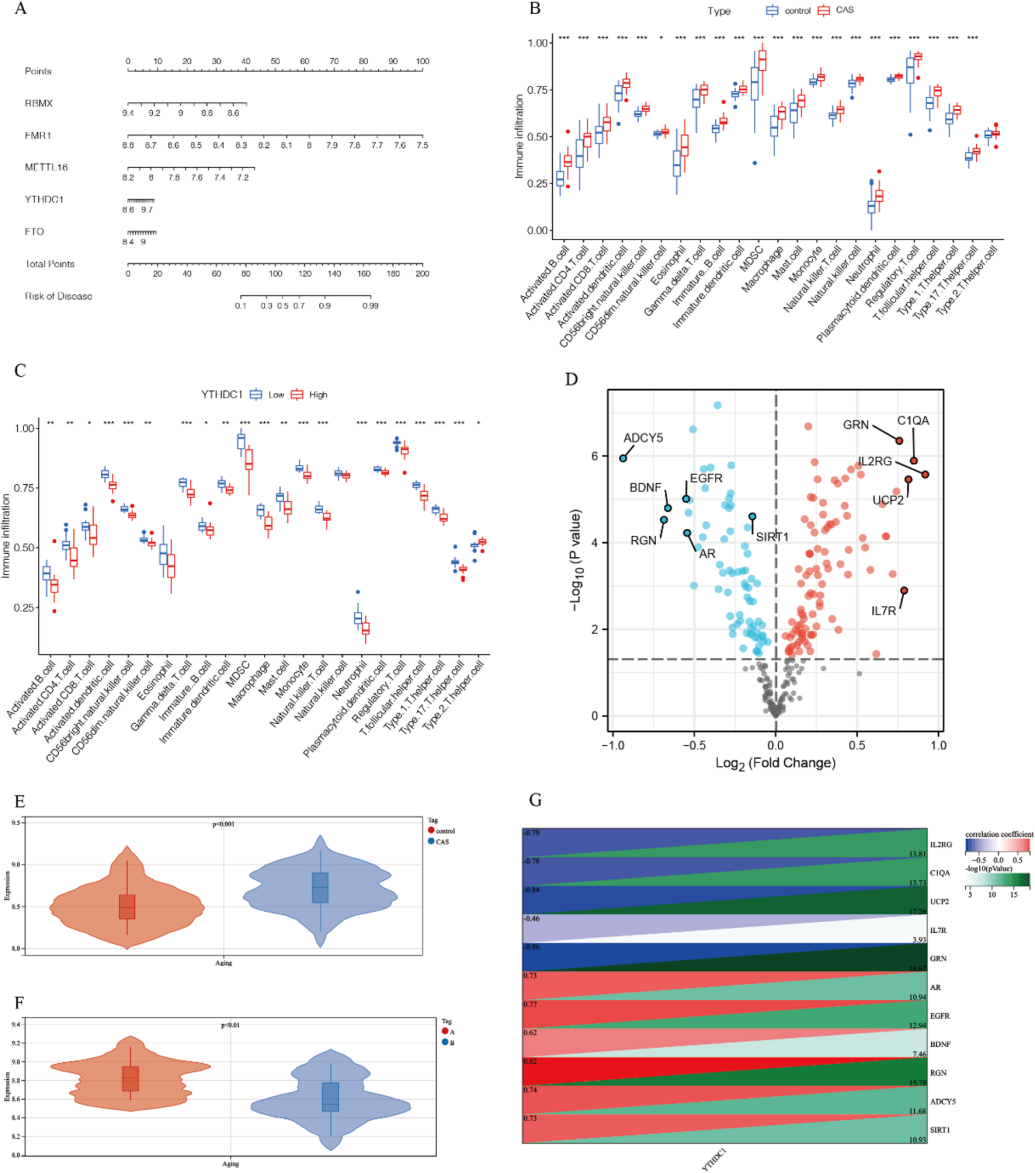
(**A**) Nomogram plots for 5 m6A regulatory factors in CAS. (**B**) Immune cell infiltration in carotid plaque and normal samples. (**C**) Relationship between YTHDC1 expression and immune cells. (**D**) Volcano plot depicting the expression of aging-related genes. (**E**, **F**) Expression of aging-related genes in normal samples and CAS samples, as well as in early and late-stage CAS samples. (**G**) Heatmap showing the correlation between YTHDC1 and aging-related genes. *P < 0.05; **P < 0.01; ***P < 0.001.

To further explore the role of m6A regulatory factors in CAS, we conducted functional enrichment analysis and KEGG pathway analysis. The results demonstrate that m6A-related genes are associated with processes such as cholesterol metabolism, oxidative phosphorylation, extracellular matrix composition, immune response regulation, and vascular smooth muscle proliferation. These processes are closely related to atherosclerosis, reaffirming the significant role of m6A modification in atherosclerosis (Fig. 3B). Subsequently, we established models for m6A regulatory factors using two machine learning methods, SVM and RF models. Figure 3C and Fig. S1C illustrate that the RF model exhibited the lowest residuals. Comparing the SVM model, the ROC curve (Fig. 3D) showed a high AUC value in the RF model. Hence, we considered the RF model as the superior model. In the RF model, the top five genes in terms of importance are RBMX, FMR1, METTL16, YTHDC1, and FTO (Fig. 3E). Subsequently, we validated the predictive model, and the results showed that the accuracy curve, ideal curve, and bias correction curve of the predictive model were highly consistent (Fig. S1E), indicating the high accuracy of the predictive model. Clinical impact curve analysis and decision curve analysis results demonstrated that the m6A predictive model can well predict the prognosis of CAS patients and can assess the net benefits of whether CAS patients need treatment and the treatment they receive (Fig. 3F, Supplementary Fig. S1F). We constructed a Nomogram based on the predictive model composed of the five genes: RBMX, FMR1, METTL16, YTHDC1, and FTO, it can better predict the risk of CAS (Fig. 4A).

### The relationship between m6A regulatory factors and immune cell infiltration and aging

Firstly, we analyzed the differences in immune cell expression between CAS and control group. All immune cells showed increased expression in CAS, with MDSCs, macrophages, activated CD4 T cells, and eosinophils showing larger differences (Fig. 4B). Secondly, we analyzed the relationship between the m6A model genes and immune cells. Low expression of YTHDC1 showed more immune cell infiltration, especially MDSCs and macrophages (Fig. 4C), it consistent with previous results. While the results for immune infiltration of the other factors can be found in Fig. S2A–D.

To explore the relationship between m6A modification and cellular aging in CAS, we confirmed the high expression of aging scores in CAS by analyzing the expression of 307 aging-related genes (Fig. 4E). Furthermore, we analyzed the differential expression of aging-related genes in CAS and the control group. The most differentially expressed aging-related genes are C1QA, IL2RG, GRN, UCP2, IL7R, ADCY5, RGN, BDNF, EGFR, AR, SIRT1(Fig. 4D). Next, we evaluated the aging scores in cluster A (low m6A expression) and cluster B (high m6A expression). The results showed that cluster A had higher aging scores compared to cluster B, indicating that the decreased expression of m6A-related genes is associated with more cellular aging (Fig. 4F).Correlation analysis was performed between YTHDC1, the regulatory molecule associated with high immune cell infiltration phenotype, and differential AG genes. The results showed that YTHDC1 had the highest correlation with RGN (correlation coefficient = 0.82) (Fig. 4F). Therefore, we speculated that the lower expression of YTHDC1 might increase the expression of inflammatory factors and lead to a decrease in the expression of the senescence-associated gene RGN.

### Single-cell sequencing reveals epigenetic changes of m6A genes in atherosclerosis

**Figure 5:**
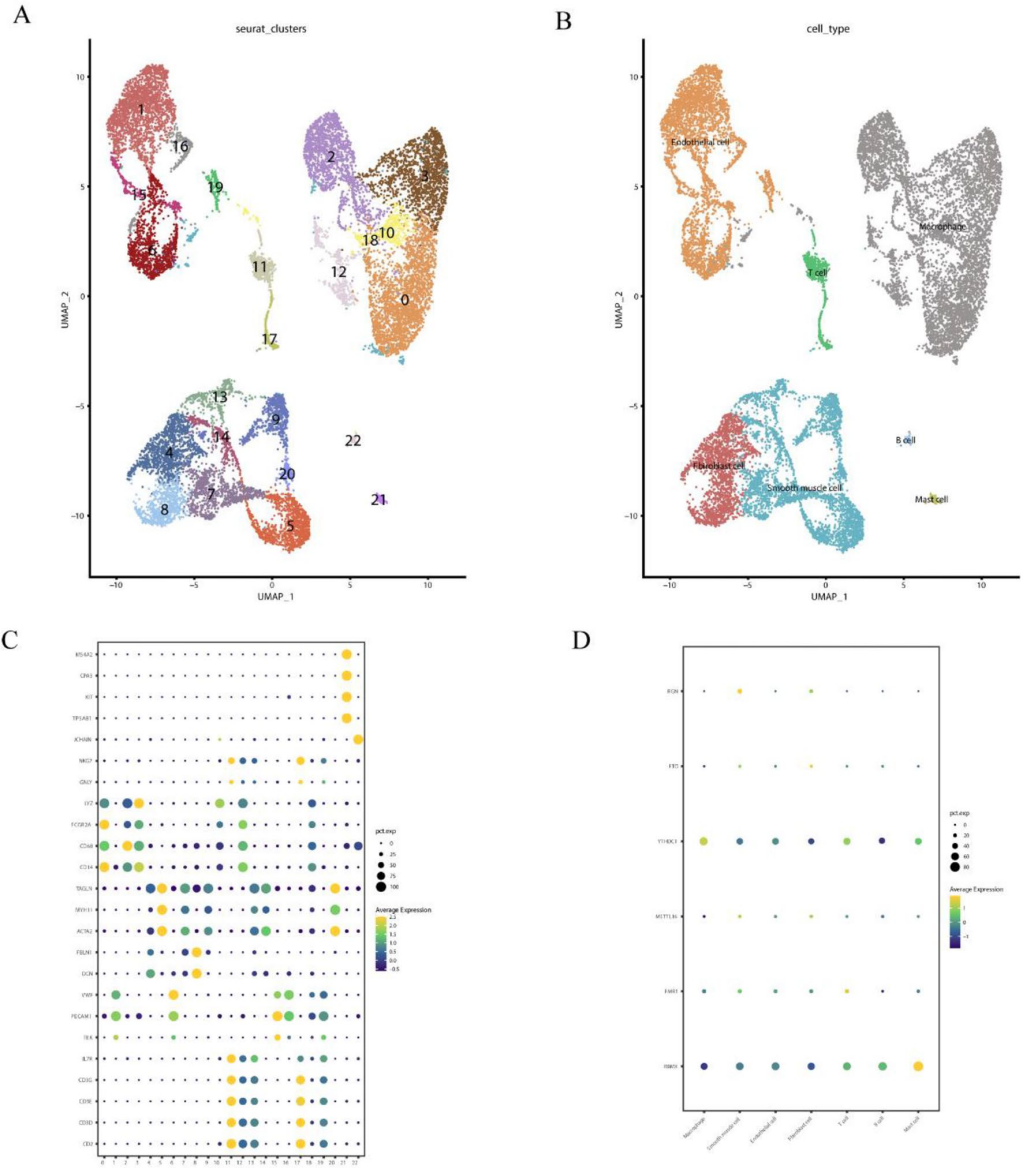
(**A**) Single-cell sequencing cluster analysis of carotid artery plaques. (**B**) Cell annotation. (**C**) Expression of relevant cell markers. (**D**) Expression profiles of m6A-related genes in different cell types.

To delve deeper into the role of m6A-related genes in atherosclerosis, single-cell sequencing data from three carotid artery plaques and the surrounding tissues were analyzed. Following quality control procedures, a total of 48,292 cells were obtained. Subsequently, these cells underwent clustering, resulting in the identification of 23 clusters (Fig. 5A). Based on relevant markers, the cells were categorized into the following groups: T cells (CD2, CD3D, CD3E, CD3G, TEK, IL7R); NK cells (GNLY, NKG7); endothelial cells (PECAM1, VWF); fibroblasts (DCN, FBLN1); vascular smooth muscle cells (ACTA2, MTH11, TAGLN); macrophages (CD14, CD68, FCGR2A, LYZ); B cells (JCHAIN); mast cells (TPSAB1, KIT, CPA3, MS4A2) (Fig. 5B,C). It was observed that RBMX exhibits high expression in mast cells, FMR1 demonstrates the highest expression in T cells, while METTL16 and FTO display elevated expression in vascular smooth muscle cells and fibroblasts. Additionally, in comparison to other cell types, YTHDC1 shows the highest expression in macrophages (Fig. 5D). Our previous findings have underscored the pivotal role of YTHDC1 in carotid atherosclerosis. Consequently, further analysis was conducted to explore the function of YTHDC1 in macrophages.

**Figure 6:**
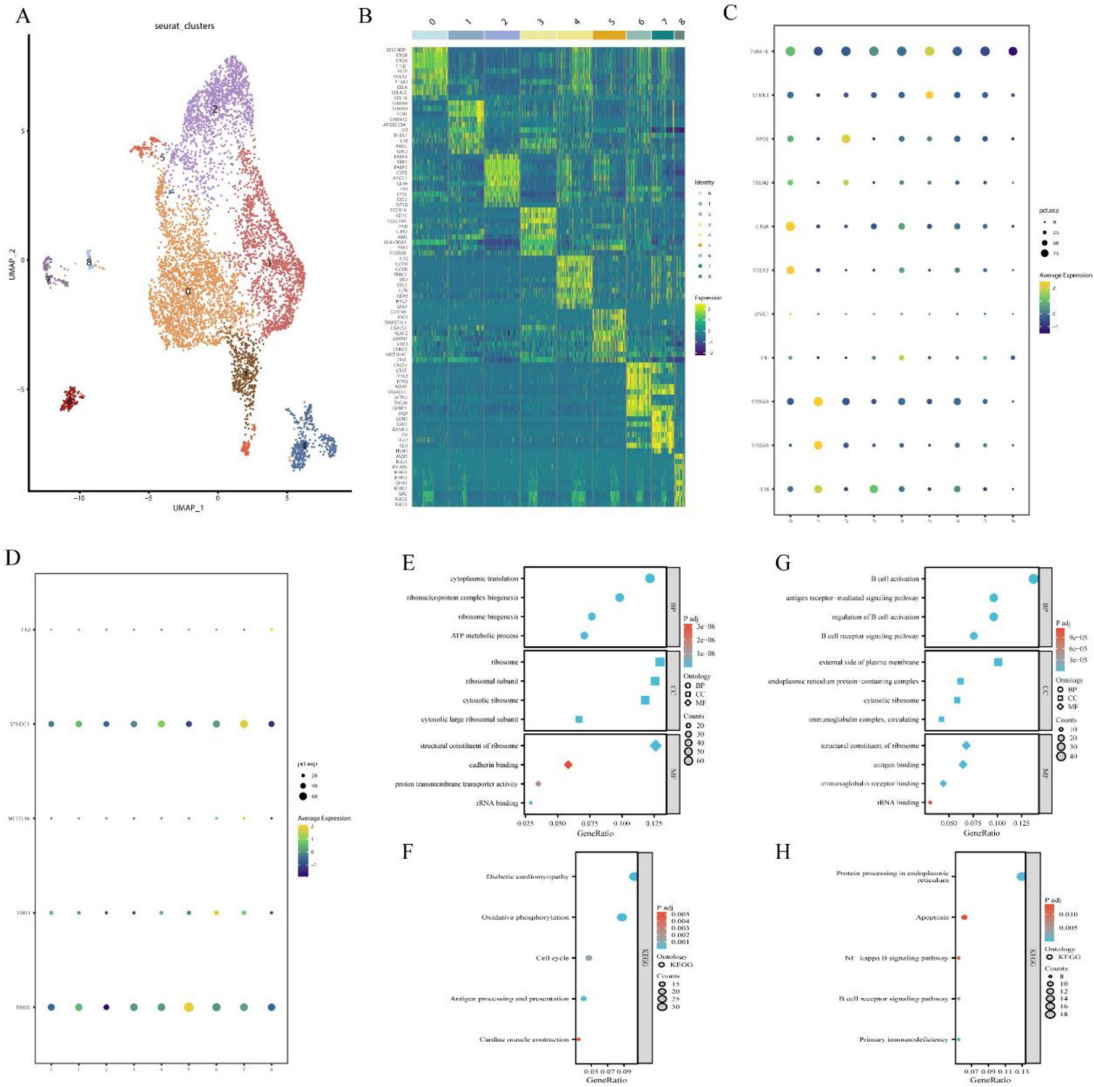
(**A**) Subcluster analysis of macrophage subpopulations. (B) Heatmap displaying the top genes in subclusters. (**C**) Analysis of macrophage subpopulations. (**D**) Expression profiles of m6A regulatory factors in the clusters. (**E**, **F**) Functional analysis and KEGG pathway analysis of Cluster 4. (**G**, **H**) Functional analysis and KEGG pathway analysis of Cluster 7.

We performed re-clustering of macrophages and obtained 9 clusters (Fig. 6A). By analyzing the top genes in each cluster and employing cell-type-specific markers for macrophage subtypes, these macrophage clusters were provisionally categorized into the following groups: Inflammatory macrophages (clusters 1, 3, 4, 6, 7), Resident-like macrophages (cluster 0), Foam macrophages (cluster 2), and Proliferating macrophages (cluster 5) (Fig. 6B,C). In Fig. 6D, it was observed that YTHDC1 displays high expression in clusters 1 and 7, both of which belong to the Inflammatory macrophage category. Furthermore, Gene Ontology (GO) and KEGG pathway enrichment analyses revealed that clusters 1 and 7 are associated with immune regulation, oxidative phosphorylation, cell cycle, and the NF-KB pathway. It was worth noting that YTHDC1 was recognized for its role in regulating the degradation rate of mRNA, thereby influencing the stability and degradation of specific genes. Among the genes significantly correlated with YTHDC1, RGN standed out, as it exhibited high expression in vascular smooth muscle cells (Fig. 5D). RGN was part of the gene group encoding calcium-binding proteins and is implicated in various cellular processes, including cell proliferation, apoptosis, cell signaling, and antioxidation, contributing to cellular defenses against oxidative stress.

Based on these findings, a hypothesis was formulated: the reduced expression of YTHDC1 in carotid atherosclerotic plaques may diminish the degradation of inflammatory factors in macrophages, thereby prolonging the inflammatory response. Additionally, the inflammatory factors released by macrophages stimulate vascular smooth muscle cells, resulting in decreased RGN expression, which, in turn, impacts cell proliferation and apoptosis, ultimately promoting the progression of atherosclerosis.

## Discussion

CVD has emerged as a significant health concern, with its incidence rising in tandem with age. It is widely recognized that both the aging process and inflammation play pivotal roles in the development of atherosclerosis, a key risk factor for CVD^1^. Atherosclerotic plaques are characterized by lipid accumulation in the arterial wall, along with infiltration of immune cells such as macrophages, T cells, and mast cells, with a fibrous cap composed mainly of collagen. In the early stages, the lesion consists of subendothelial lipid deposition and infiltration of cholesterol-loaded macrophage foam cells and T cells. When lesions progress to advanced stages, necrotic cells, apoptotic cells, cellular debris, and cholesterol crystals form a necrotic core in the lesion^26^. m6A RNA methylation has emerged as one of the most prevalent internal RNA modifications and has been associated with a spectrum of human diseases^27^, encompassing CVD^28–30^, chronic obstructive pulmonary disease^31^, neurodegenerative disease^32^, cancers^33^, and metabolic syndromes^34^. Recent research has highlighted that m6A-related genes play a role in numerous biological processes associated with carotid atherosclerosis^35^. However, the exact exploration of m6A modification in the context of carotid atherosclerosis and its correlation with the immune microenvironment and aging characteristics have to be studied more systematically.

Here, we uncovered the interactions between altered transcript levels and m6A regulators in carotid atherosclerosis, first of all, we found 7 m6A regulators that were significantly altered in both datasets, suggesting that m6A regulators are strongly involved in the development of CAS. In addition, we found significant differences in immune cell ratios between individuals with and without carotid atherosclerosis, indicating that carotid atherosclerotic samples were highly immunoreactive compared with healthy controls, with high infiltration of T cells, B cells, mast cells, macrophages, and neutrophils consistent with atherosclerotic plaque lesions. We further compared the differences in the immune microenvironment of different m6A regulatory factor modification models and showed that cluster A had more immune cell infiltration.

Then, GO and KEGG pathway analysis of m6A regulators implied that these regulators might be involved in cholesterol metabolism, PPAR signaling pathway, smooth muscle cell proliferation, positive regulation of MAPK cascade, inflammatory response and so on. The patients with hypercholesterolemia and hypertension are at high risk for AS, and long-term use of antihypertensive and lipid-lowering drugs is the routine treatment for CAS^36^. As for the inflammation in atherosclerosis, it normally comes from various subpopulations of macrophages, T-lymphocytes and mast cells. Clinically, many patients can slow down the development of AS by taking antihypertensive drugs and lipid-lowering drugs, however, there is nevertheless a risk of cardiovascular events, which may be attributed to unstable plaques caused by unresolved inflammation^37^. The PPAR signaling pathway is participated in a wide variety of biological processes, including oxidative stress, apoptosis, and lipid metabolism^38^. By modulating the PPAR signaling pathway might be beneficial for AS patients. With these analyses, we believe that m6A regulators may be involved in more than one role during CAS development.

In search of more critical m6A regulators, we constructed models with m6A regulators by machine learning, which indicated that the Random Forest (RF) model has a considerable advantage. Nomogram provides a new avenue for in-depth analysis of patients with carotid atherosclerosis. It provides a user-friendly interface to assess the risk of carotid atherosclerosis based on collected patient blood samples. By measuring the expression levels of five identified biomarkers, a score is given for each gene, and the sum of these scores is the total score, which can be used to predict the likelihood of developing carotid atherosclerosis. In analyzing the five m6A regulators for differences in immune cell abundance between early and late CAS samples, we found that YTHDC1 had a better correlation with immune cell infiltration than the other regulators. Existing studies have shown that physiological high shear stress reduces the mRNA and protein levels of ras homolog family member J in endothelial cells, thereby reducing the inflammatory response of endothelial cells, with the involvement of YTHDC1^39^. Furthermore, YTHDC1 can regulate the apoptosis of pulmonary artery endothelial cells by reducing the stability of FENDRR^40^. In addition, excessive inhalation of PM2.5 increases the expression of METTL16 in rat lung microvascular endothelial cells and induces microvascular damage by modifying the expression of Sulf2^41^. Furthermore, FTO has been found to have a significant impact on vascular function, including inhibiting the release of vascular endothelial growth factor and angiogenesis, counteracting metabolic and vascular dysfunction caused by obesity, and inducing proliferation and inflammatory response of vascular smooth muscle cells^42–44^.

There is growing evidence that aging is also a major risk factor for promoting atherosclerosis^45,46^. The senescent endothelial cells and vascular vessel smooth muscle cells not only have a weakened protective capacity, but also promote the expression of pro-inflammatory factors, exacerbate vascular inflammation, and to a certain extent, increase the uptake of plasma lipoproteins. More importantly, cellular senescence exacerbates apoptosis in endothelial and smooth muscle cells, which has been confirmed by many studies. This is the first study to explore the link between m6A regulators and aging-related genes in CAS. After screening we found that many aging-related genes may be affected by m6A regulatory factors, among which YTHDC1 and RG were significantly associated. Finally, we performed single-cell sequencing analysis of 3 carotid plaques and surrounding tissues, which showed that YTHDC1 had the highest expression in macrophages and was associated with inflammatory processes. Correspondingly, RGN was significantly expressed in vascular smooth muscle cells. Accordingly, we consider that low expression of YTHDC1 leads to elevated RGN expression by promoting macrophage inflammation, which increases apoptosis in vascular smooth muscle cells and ultimately exacerbates atherosclerosis. Therefore, we suggest that low expression of YTHDC1 leads to decreased RGN expression by promoting macrophage inflammation, resulting in increased apoptosis in vascular smooth muscle cells. This ultimately exacerbates atherosclerosis.

Several limitations are inherent in our study. Firstly, we attempted to identify additional datasets for model validation and clustering analysis but were unable to locate suitable datasets beyond the GEO database. Secondly, the atherosclerotic plaques obtained from surgical patients may represent the terminal stage of lesion evolution. Thus, a more comprehensive analysis of immune components within plaques at various stages is warranted to capture the dynamic changes in the plaque's immune environment more thoroughly. Further and more refined investigations are required.

## Conclusions

In summary, a reduced level of m6A methylation modification could enhance inflammation and expedite cellular aging, thereby contributing to the development of carotid atherosclerosis.

### Supplementary Information

Supplementary Figures.

## Data Availability

The dataset generated and analyzed during the current study is available in the Gene Expression Omnibus (GEO) under accession number GSE43292, GSE28829 and GSE159677 (https://www.ncbi.nlm.nih.gov/geo/).
