# Supplementary material for: M6A plays a potential role in carotid atherosclerosis by modulating immune cell modification and regulating aging-related genes
Source: Sci Rep. 2024 Jan 2;14:60. doi: 10.1038/s41598-023-50557-8 (PMC10761844; doi:10.1038/s41598-023-50557-8)
Supplement: Supplementary file 1 — Supplementary Figures. [file 41598_2023_50557_MOESM1_ESM.docx]

M6A plays a potential role in carotid atherosclerosis by modulating immune cell modification and regulating aging-related genes

Wenpeng Zhao ^1,^ †,Yinqi Xu^1,^ †, Jiabao Zhu ^1^, Chaoxuan Zhang ^2^ , Weimin Zhou ^1,*^and Shizhi Wang ^1,^*

|  |
| --- |

^1^ Department of vascular surgery，the Second Affiliated Hospital of Nanchang University, No. 1 MINDE ROAD, NANCHANG, 330006, JIANGXI PROVINCE, CHINA, 330006

^2^ Queen Mary College, Nanchang University, Nanchang, Jiangxi, China, 330031

***** Authors to whom correspondence should be addressed.

Correspondence: Weimin Zhou email：[zwmsubmit@126.com](mailto:zwmsubmit@126.com), Shizhi Wang email：[119456283@qq.com](mailto:119456283@qq.com)

† These authors contributed equally to this work


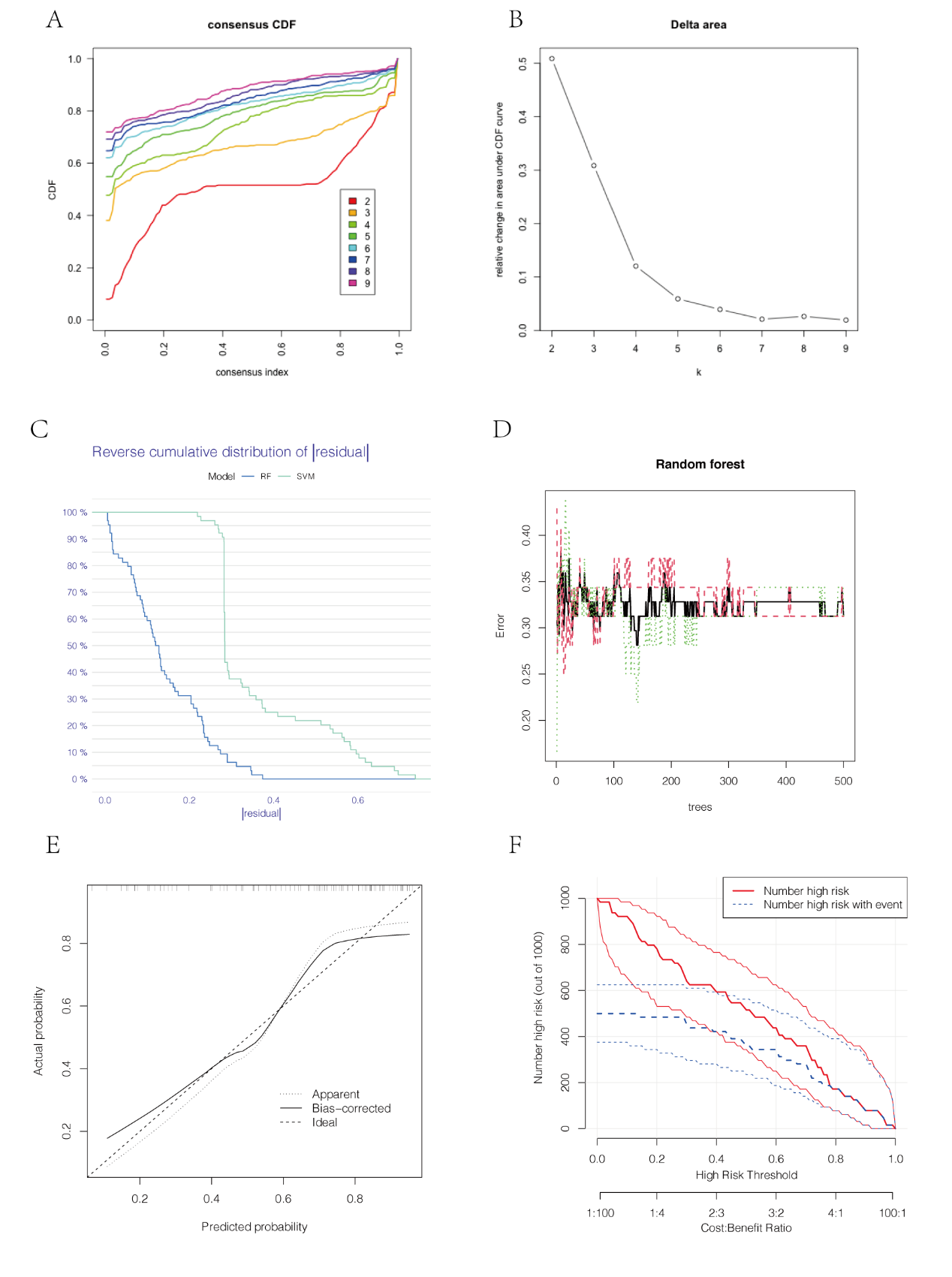


**Figure S1.** (A, B) CDF curves, based on copynetic value, with the best rank being 2. (C) Residual curves of the RF and SVM models. (D) Error plot of the Random Forest (RF) model. (E, F) Calibration curves for predicting CAS in the samples and actual CAS. (F) Clinical impact curves of the models.


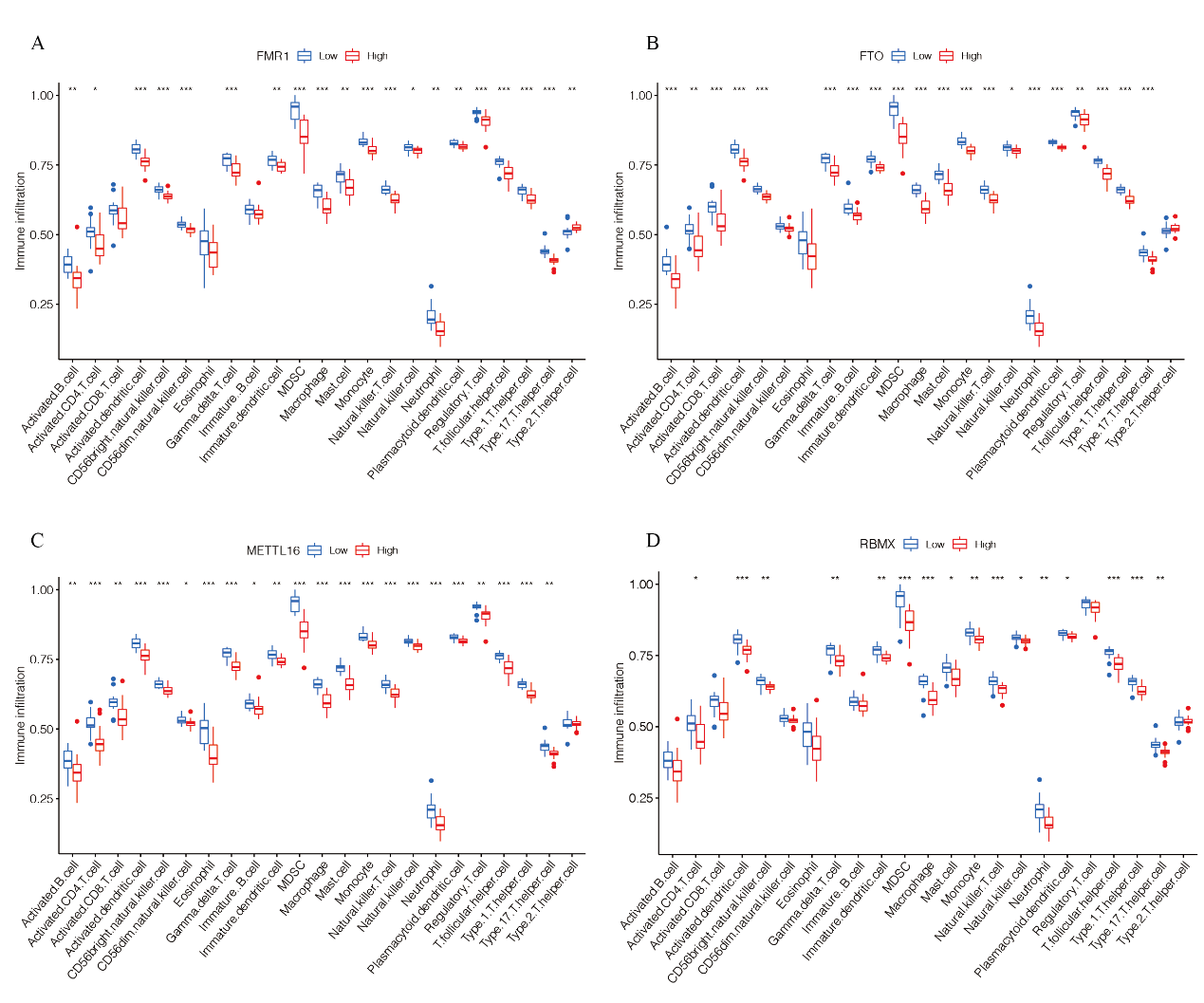


**Figure S2.** The immune microenvironment of the remaining four m6A regulatory factors in early-stage and late-stage CAS.
